# Human–machine teaming is key to AI adoption: clinicians’ experiences with a deployed machine learning system

**DOI:** 10.1038/s41746-022-00597-7

**Published:** 2022-07-21

**Authors:** Katharine E. Henry, Rachel Kornfield, Anirudh Sridharan, Robert C. Linton, Catherine Groh, Tony Wang, Albert Wu, Bilge Mutlu, Suchi Saria

**Affiliations:** 1 Department of Computer Science, Johns Hopkins University, Baltimore, MD USA; 2 Department of Preventive Medicine, Feinberg School of Medicine, Northwestern University, Chicago, IL USA; 3 Center for Behavioral Intervention Technologies, Northwestern University, Chicago, IL USA; 4 Howard County General Hospital, Columbia, MD USA; 5 Department of Industrial Engineering, University of Wisconsin-Madison, Madison, WI USA; 6 Department of Health Policy and Management, Johns Hopkins Bloomberg School of Public Health, Baltimore, MD USA; 7 Department of Computer Sciences, University of Wisconsin-Madison, Madison, WI USA; 8 Department of Applied Mathematics and Statistics, Johns Hopkins University, Baltimore, MD USA; 9 Bayesian Health, New York, NY 10005 USA

**Keywords:** Outcomes research, Translational research, Diagnosis

## Abstract

While a growing number of machine learning (ML) systems have been deployed in clinical settings with the promise of improving patient care, many have struggled to gain adoption and realize this promise. Based on a qualitative analysis of coded interviews with clinicians who use an ML-based system for sepsis, we found that, rather than viewing the system as a surrogate for their clinical judgment, clinicians perceived themselves as partnering with the technology. Our findings suggest that, even without a deep understanding of machine learning, clinicians can build trust with an ML system through experience, expert endorsement and validation, and systems designed to accommodate clinicians’ autonomy and support them across their entire workflow.

By taking into account numerous patterns of risk and leveraging a wide variety of data sources, machine learning (ML) has the potential to improve upon rule-based clinical decision support systems (CDSS) in supporting clinical care, even identifying patterns not apparent to human experts^1^. However, the impact of ML systems in medicine depends on clinicians consulting them and applying their insights. Integrating ML could present a challenge in time-constrained clinical contexts, where clinicians must rapidly evaluate whether and how to act on recommendations while managing competing demands on their time and attention^2,3^. Indeed, most systems typically report users responding to only 6–45% of alerts or requiring dedicated staff^4–6^. It is not yet well-understood how clinicians perceive ML recommendations in these contexts, and thus it remains unclear how to most effectively deploy ML systems to maximize clinical benefit^7–9^.

Two predominant hurdles have been theorized that potentially compromise clinicians’ willingness to integrate ML models into their work. First, experts may struggle to develop trust with ML-based systems due to a large number of inputs and the complex integration of data involved, which can make it challenging or impossible to convey the specific logic behind an alert or recommendation^10–12^. Second, some evidence suggests that many view ML as being impoverished relative to human expertise and question whether it can add clinical value for highly trained expert users^13,14^. By making a competing diagnosis, ML systems could also be perceived as encroaching on clinicians’ professional role, presenting a “threat to autonomy” that may make clinicians reluctant to use, rely on, and trust them^15,16^.

The purpose of this study is to understand (1) the role that clinicians see ML as playing in acute clinical care, and (2) pathways and barriers to building trust with ML-based recommendations. Through a series of in-depth interviews with 20 physicians and nurses, analyzed following a grounded theory approach (see Methods), we explore these issues in the context of a deployed ML-based system: the Targeted Real-time Early Warning System (TREWS) for sepsis, a syndrome with high mortality and morbidity wherein a whole-body inflammatory response causes organ dysfunction^17–20^. Leveraging an ML-based risk score, TREWS was developed to support clinicians’ timely identification and treatment of sepsis (See Methods for system description)^21,22^. Whereas most ML systems have not yet been broadly deployed and adopted in clinical practice^23–25^, TREWS was integrated into the electronic health record (EHR) at a large non-teaching hospital beginning in 2017 and was in use for over 6 months at the time of this study, with high, sustained adoption^21^. At a moment when ML-based support is poised for broad deployment across the healthcare system, this work seeks to inform system designers, implementation scientists, clinicians, and healthcare administrators by clarifying the experiences and views of clinicians tasked with using this emerging technology in their day-to-day work.

A Grounded Theory analysis was systematically applied to transcripts from the interviews to identify themes characterizing how an ML system is perceived and used by clinicians in practice (Supplementary Table 1), yielding four primary themes described below.

The first theme identified was that, while clinicians appreciated that the ML-based system improved upon other clinical support systems, they generally did not differentiate the operations between ML-based and conventional CDSS. As far as comparing the ML-based system to prior CDSS they had used, most clinicians recognized its improved reliability and timely information. Indeed, all clinicians favorably compared TREWS to the rule-based CDSS that had previously been in place for sepsis. That alert, based on meeting at least two of the SIRS criteria and an indicator of organ dysfunction^26^, was perceived as having low precision, and as repeatedly alerting the same patient when a diagnosis was already clear, preventing clinicians from developing trust in the system and leading to some frustration. For instance, one physician described the previous system as “irritating” because “it kept telling you to reorder lactate every time you’d open the patient’s chart,” even if the provider had previously indicated that the patient did not have sepsis.

However, despite recognizing these improvements, clinicians lacked an understanding of how specifically the ML-based system achieved higher reliability than the prior CDSS. While describing the model behind the ML-based system as being generally more “sophisticated” than a conventional CDSS, most could not identify how the internal logic of the ML-based system differed from that of rule-based CDSS, often attributing improvements solely to the fact that the ML-based system considers a larger number of measurements. Others appeared to have an incorrect understanding of the ML-based model, assuming that it (like the prior CDSS) simply checked whether parameters exceeded established thresholds. In actuality, instead of using prespecified thresholds, the ML behind the system learned multidimensional indicators of risk from the data, combining patient history and clinical presentation into a predictive risk score. However, as the next section describes, this limited understanding of the ML system’s operation was not a substantial barrier to use.

The second theme identified from the interviews was that clinicians perceived the ML-based systems as playing a supporting role both in and beyond diagnosis. Regardless of their understanding of the ML behind the system, physicians were generally responsive to its alerts and integrated them into their diagnostic process. However, they saw themselves as maintaining ultimate responsibility for diagnosis and treatment decisions. Thus, physicians acknowledged that an alert might “make you think of [an] alternative diagnosis” but this was differentiated from “swaying” or “influencing” the physician. In cases where they disagreed with the system recommendation (sepsis or not), physicians reported that they would rely on their own judgment. Therefore, while alerts led to increased consideration of cases highlighted by the system, and required incorporating new information into decision-making, they were not seen as disrupting clinicians’ agency.

Clinicians also differentiated their diagnostic process from the capabilities of ML, emphasizing elements of clinical expertise and intuition that they felt ML could not replicate. Multiple providers referenced the visible cues and richer information available from interacting with the patient at the bedside. An ED physician expressed, “[The system] can’t help you with what it can’t see.”

Clinicians also found value in the ML-based system beyond the point of diagnosis. Both nurses and physicians reported viewing the system as a “second pair of eyes” to bring cases to their attention or to alert them to a change in the patient’s state. This ongoing monitoring by the system was experienced as alleviating some demands on their attention and cognition in a context of feeling “bombarded with clinical information.” One physician described the importance of “anything that helps cognitive unloading.” They additionally described the system as helping to prompt time-dependent actions and to coordinate across multiple care team members. In the ED, the system helped both physicians and nurses prioritize which patients to see next. A nurse described, “I think we try to get them in front of a provider a little bit quicker or get some of the stuff started out in triage.” Support for non-diagnostic tasks added to the overall perceived value of the ML-based system.

The third theme was that clinicians identified a variety of mechanisms they used to establish trust with the system. Clinicians described that their overall willingness to trust the ML-based system was rooted in several factors that helped them build a mental model of how the system worked. None of the clinicians fully understood the machine learning behind the system; however, while some were curious to learn more, they did not perceive that understanding the system’s logic in an individual case would change their decision-making. One physician described, “For clinicians, I think just understanding [that] this is a machine learning tool and it does data mining, I think will be more than enough.” Although unconcerned with the specific statistical model behind the system, clinicians reported having come to better understand how the system operated by observing its behavior in different scenarios and with different patient types.

In addition to relying on direct experience of the system, clinicians valued external studies of the system and recommendations by colleagues and experts that allowed them to develop trust peripherally. Many clinicians described using the system because a colleague or department head had endorsed it, or they had seen descriptions of the system’s development and validation process. For instance, one ED physician said, “I’d want to understand the population it was derived from… and then I’d want to see the population that they validated it on afterwards… whether that group looks like the patients that I’m treating.”

Clinicians also valued that they were able to ask questions about system design choices during educational sessions and customize the interface and alert sensitivity to their environment and patient population. One described, “I need to understand the motivation behind that tool because when I apply that tool, I’m applying the judgment of the creators of that tool.” Interacting with the deployment team also allowed for input into the tool’s operations, which was described as an improvement over prior CDSS deployments.

Finally, the fourth theme identified from the interviews was the remaining perceived barriers to the use of ML in medicine. While clinicians were generally enthusiastic about the potential for ML-based systems to improve patient care, they expressed some concerns. Several pointed to the potential for over-reliance on automated systems, which could ultimately degrade their clinical abilities: “I think [that] there are a lot of people, frankly, that will quickly default to having a tool tell them what to do and stop assessing, and I hope that’s not true, but I’ve seen it happen.” Several also mentioned concerns that regulatory agencies might use these systems to standardize care even in scenarios where a clinician disagrees with the system, potentially leading to over-treatment and patient harm, especially in cases where the alerts occurred prior to clinical recognition. When asked what would convince them to act on the system’s recommendations prior to apparent symptoms, suggestions included clinical trial evidence, or personally experiencing scenarios where the alert was dismissed but the patient was later diagnosed as having sepsis.

Ultimately, the capacity of state-of-the-art ML systems to improve clinical care depends on clinicians’ ability and willingness to incorporate the information provided by these systems into their work. A Grounded Theory approach, systematically applied to text data from interviews with 20 clinicians using an ML-based system as part of routine clinical practice in an acute-care setting, identified themes related to the use and perception of ML-based clinical support systems. Key findings included (1) that clinicians at the bedside did not perceive interpretability, in the sense of understanding the calculations of the ML model that led to a specific patient’s recommendation, as a primary driver of their use of the system, (2) that they viewed the system as augmenting both their diagnostic and treatment management processes rather than supplanting their clinical judgment, (3) that they developed a mental model or understanding of how to leverage the system both through direct observations and indirectly through interactions with peers, research team members, and empirical studies, and (4) that some barriers remain to their trusting ML in medicine. Below, we outline the implications of these findings for the design and implementation of systems that use ML to support clinical work.

A growing body of recent work has raised questions about whether physicians would be willing to accept ML-based recommendations in the absence of understanding the underlying ML model^27–29^. Contrary to our expectations, our findings suggest that interpretability of the model’s computations was not perceived as a barrier among these clinicians. Instead, numerous factors play a role in shaping how clinicians perceive and act on ML recommendations, with many of the most influential mechanisms of trust occurring outside the timepoint when a clinician evaluates a specific TREWS recommendation. Prior work has found that time constraints prevented primary care providers from engaging deeply with a ML-based treatment recommender system and that trust was better established outside of in-the-moment use^12^. Similarly, we found that competing time demands limited clinicians’ ability to explore advanced features of the system, such as feature importance measures. While future work is needed to clarify how systems might more efficiently communicate feature importance to build trust during time-constrained clinical interactions, our findings suggest alternative pathways through which trust can be built outside of patient care, including through adequate onboarding of users, training, and technical support (e.g., a technology “navigator”)^30^, discussions among colleagues who have used the system and sharing findings from relevant studies. In addition, many clinicians pointed to ongoing relationships with the development team during the onboarding process as allowing for refinements of the tool to respond to their needs. This involvement in early system development may go against the norms for CDSS deployment and may have contributed to clinicians’ receptivity to the tool^31^. The implementation science literature likewise suggests the importance of early feedback to align an intervention to its eventual setting^32^.

Much recent work evaluates how well ML-based systems perform as a surrogate for clinician judgment, which has also led to concern that clinicians will view these systems as inherently threatening to their professional authority and avoid adopting them^15^. While clinicians described the current ML-based system as more reliable than the previous system and valued that it could learn from past interactions, our results highlight that, in practice, the ML-based system is still not seen as having the ability to match clinicians’ judgment, and is therefore not perceived as a threat. Rather than a tool or form of automation that might supplant a specific function, clinicians described that the tool *partnered* with them, e.g., by drawing their expert attention to patients who needed it in a timely fashion. The system was therefore viewed as augmenting a decision-making process that remains fundamentally clinician-directed. This view parallels recent work that has proposed “teaming” between intelligent machines and humans, where each leverages the strengths of the other to achieve better outcomes^33–35^. System designers should continue to focus on augmenting rather than replacing clinician judgment in order to maintain adoption and protect against over-reliance.

Evaluations of ML-based systems for deployment often emphasize identifying patients as early as possible. However, our data indicate that physicians highly valued direct observation of the patient to validate a recommendation by the system, and this ability to directly observe the patient may have increased their willingness to use alerts from a ML-based system. As current provider practices involve the identification of specific symptoms to trigger treatment guidelines, predictions that are too early for the provider to validate may therefore result in rejection of system recommendations and diminished trust. Reflecting these considerations, which were relayed to the development team in early feedback from clinicians and the hospital’s quality and safety team, the system in this study delayed alerts until verifiable symptoms were present, which likely contributed to trust and acceptance in this study. The design of systems that provide earlier predictions must consider providers’ ability to verify symptoms and, in the absence of such symptoms, identify alternative strategies to gain clinicians’ trust and adoption of early interventions. Acceptance of early prediction may be compromised if clinicians lack opportunities to appreciate the accuracy of ML-based tools, especially if they view them primarily as a cost-cutting measure, supplanting highly skilled and intuitive work with something inferior but more scalable.

The main limitation of this study is that it relies on 20 interviews conducted in a single hospital with a single ML-based system used at the bedside. While the in-depth interview format limited the number of interviews conducted, we interviewed physicians and nurses across several unit types to get a broad perspective. A follow-up quantitative study looking at 2 years of data from this and four other hospitals using the same ML-based system shows that high adoption is sustained across all sites^21^. However, the high receptivity to the system observed here may not be typical across deployments of new technologies. Future work is needed to assess how these findings generalize to other hospital systems and clinical specialties where providers may have different experiences with CDSS and personal attitudes influencing their openness to new technologies. Additionally, while the qualitative approach we adopted here offers provisional theories about the use of ML-based systems in clinical decision-making, these are based on a small number of interviews at a single site and should be evaluated in follow-up studies using hypothesis-testing approaches.

## Methods

Between October 2018 and April 2019, the research team conducted semi-structured interviews with 20 clinicians who used TREWS in their daily work at a community hospital. The study protocol was approved by the Johns Hopkins Medicine Internal Review Board (IRB00252594) and informed consent was obtained from all participants. The collected interviews were then transcribed and coded in an iterative process using a Grounded Theory approach to identify themes in the interviews. See the “Data Analysis” section of the Methods for further details about the Grounded Theory approach used and example questions from the interview guide.

### Setting

This study was conducted at a 285-bed, acute-care, non-teaching hospital in the northeastern United States. At the start of the study, Targeted Real-time Early Warning System (TREWS), a machine learning (ML)-based system for sepsis detection and treatment management was in use in the emergency department (ED) for 11 months and across all medical and surgical units, including the intensive care unit (ICU), at the hospital for 6 months at the start of study enrollment. We describe the system in further detail below. Prior to TREWS, the hospital had used a rule-based sepsis best practice alert (a type of CDSS) that was built into the Epic EHR environment and that generated a pop-up alert for sepsis whenever a patient met at least two of the criteria for systemic inflammatory response syndrome and one of the organ dysfunction criteria specified by the Centers for Medicare and Medicaid sepsis core measure (SEP-1)^26^. This rule-based alert was turned off following the deployment of TREWS.

### System description

Developed from 5 years of historical data collected from three hospitals, TREWS uses an ML approach to learn patterns from time-series data to predict, in real-time, whether a patient is at risk of developing sepsis^21,22,36,37^. In order to account for the heterogeneity of patients with sepsis, the risk prediction method automatically discovers multiple phenotypes of sepsis and learns from provider behavior over time to improve sequential predictions. It also reduces false positive alerts, thus improving precision, by accounting for confounding comorbidities that can cause automated systems to mistakenly identify a patient as having sepsis^21,38^. Based on provider feedback, the version of TREWS deployed at this hospital waited for an indicator of organ dysfunction prior to alerting. Evaluated on 2 years of data from three community and two academic hospitals (469,419 screened encounters), the system generated 31,591 alerts, 89% of which had an evaluation entered within the system page (53 and 73% of alerts had an evaluation entered within 1 and 3 h, respectively)^21^. The system had a sensitivity of 82%, with a corresponding 38% of evaluated alerts confirmed as having sepsis^21^. While we were unable to directly compare performance to the prior system due to lack of alert records, evaluations of similar alert criteria at other hospitals have found that such criteria have low precision, with fewer than 20% of patients with an alert having sepsis^39^. The precision of rule-based CDSS is generally even lower during deployment since there can be multiple false alerts on the same patient^40^.

### Interface description

The TREWS interface is a dynamic system that adapts based on the type of user and patient status. It consists of three main components: a nursing assessment section, a provider evaluation section, and a treatment management section. While all components are visible to all users, actions on the page are user-specific and aligned with the scope of their clinical practice (e.g., only providers are able to place orders through the treatment management section). After an alert is activated, providers are requested to complete an evaluation within the TREWS page to either confirm that the patient has sepsis, dismiss the alert, or temporarily pause the alert until more information is available (Supplementary Fig. 1). In order to help explain the factors contributing to an individual alert, the TREWS page also contained a list of the measurement names and values that were included in the model and starred the features that were considered most important. We computed a feature’s importance to the model based on ranking the magnitude of change in the predicted risk if that value were replaced with the population mean value. However, a numeric ranking of features was not shown to users, only a star indicating the top features.

### Deployment description

The deployment process consisted of three main phases: pre-deployment, ED-deployment, and full hospital deployment. In the first phase, the team prepared for deployment by verifying that the alert was correctly integrated with the EHR and running as expected, met with key stakeholders to identify clinical champions and discuss the integration of the alert with the clinical workflow and met with clinicians to explain the alert including how it was developed and validated, the types of information the system uses to generate alerts, and how to use the interface. The ED-deployment phase took place between November 2017 and March 2018. During this time, the system was only active in the ED in order to allow the deployment team to verify the implementation and make refinements to the model and system workflow. Starting March 2018, the deployment was expanded to all inpatient units at the hospital and entered a maintenance phase of the deployment. Throughout the ED and all hospital deployment phases, the deployment team participated in staff meetings and met with individual clinical users, as requested, to explain the system and how to use the interface. A feedback button was also implemented to allow users to ask questions and seek clarification about the system’s behavior.

### Data collection

Between October 2018 and April 2019, the research team conducted semi-structured interviews with 20 clinicians who used TREWS in their daily work at a community hospital. Physicians and nurses who had used TREWS for at least 6 months were asked to participate, thereby representing a number of units and clinical roles. Among clinicians who responded to the investigator’s interview requests and were available at the interview times, a representative sample of nurses and physicians across different unit types was selected. Interviews were conducted until saturation was reached, such that conducting new interviews failed to generate novel themes and insights. Study participants included 13 physicians (4 ED, 4 critical care, 5 general ward) and 7 nurses (3 ED, 4 critical care). Interviews were conducted at the hospital, in each participant’s work environment (e.g., nurse’s stations, private office, etc.). The interviews were conducted by one of two graduate research assistants who were familiar with the system and clinical environment and had been trained in semi-structured interview methods. The interview guide was developed collectively by all authors and questions concerned clinicians’ role in diagnosing and treating sepsis, their experience with CDSS in general, their experience with TREWS and other ML-based CDSS, and their thoughts regarding the current and future role of ML in medicine. For example, interviewees were asked questions including, “How would you describe how TREWS works to a coworker who had not used it before?”; “What impact has TREWS had on your clinical practice, if any?”; “When evaluating an alert, what information inside or outside the page do you consider?”; “Do you have any concerns about the use of intelligent systems in medical diagnosis?”; “Can you describe any ways you have used AI tools in your life outside of medicine?” The interviews were audio-recorded and then transcribed and anonymized.

### Data analysis

Inductive coding approaches are well-suited for research questions where there is limited established theory^41^. Given the limited understanding of the factors driving clinicians’ perceptions and adoption of ML, a Grounded Theory approach (a type of inductive approach) was employed for data analysis, with themes emerging from a review of the transcripts rather than determined a priori^42^. We consulted the Standards for Reporting Qualitative Research (SRQR) guidelines in presenting our process and findings^43^.

Consistent with our Grounded Theory approach, three researchers employed a process of open coding, axial coding, and selective coding, moving from less to more formalized code definitions as they narrowed their area of inquiry. The second author, who is experienced in qualitative analysis, trained the other two coders. After immersing themselves in the data by reading all transcripts, the three coders first engaged in open coding in which they coded the transcripts for a broad set of preliminary themes related to clinicians’ roles in sepsis care, experiences with ML-based tools, and perceptions of these tools and of AI. The three coders then met to discuss, name, and define these codes, resulting in a shared codebook. In an iterative process, the three coders each read and coded overlapping transcripts, and then met to refine and formalize the codebook, including adding new themes emerging from the data and adjusting code definitions to better encompass the data. Next, axial coding was performed in which researchers established connections between codes and grouped them hierarchically. For example, the axial code for “Trust in AI” included subcodes capturing “trust based on experience” and “trust based on peer recommendations.” The coders engaged in selective coding by narrowing their coding to focus on those areas of the codebook most central to understanding the use of ML, excluding more tangential areas of the codebook. Four primary themes emerged from the axial coding. As is common for Grounded Theory analyses^44^, no formal inter-rater reliability metrics were computed; however, the coding process included repeated discussions of coding discrepancies to increase consistency and arrive at a consensus. Consensus coding is designed to catch errors, reduce groupthink, and minimize researcher biases while recognizing data complexity^45^. After several rounds of iterations, additional coding failed to yield coding discrepancies or codebook revisions. After agreeing to this final codebook, the uncoded transcripts were divided among the coders.

### Reporting summary

Further information on research design is available in the Nature Research Reporting Summary linked to this article.

## Supplementary information

Supplemental Materials

Reporting Summary

## Data Availability

The full transcripts of the interviews are not publicly available in order to minimize the risk of participant reidentification. Summaries of the interview contents and related metadata that support these findings are available from the corresponding author upon reasonable request.
