# Supplementary material for: Human–machine teaming is key to AI adoption: clinicians’ experiences with a deployed machine learning system
Source: NPJ Digit Med. 2022 Jul 21;5:97. doi: 10.1038/s41746-022-00597-7 (PMC9304371; doi:10.1038/s41746-022-00597-7)
Supplement: Supplementary file 1 — Supplemental Materials [file 41746_2022_597_MOESM1_ESM.pdf]

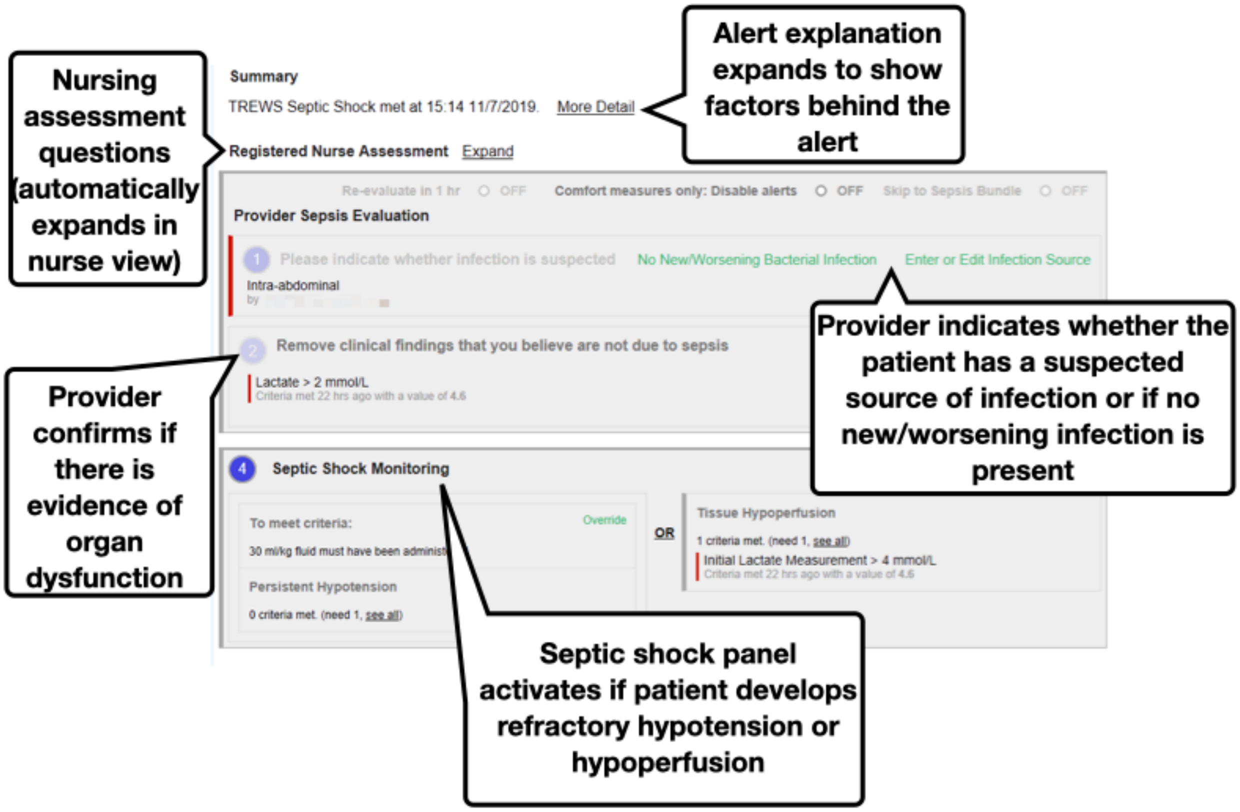

Supplementary Figure 1. Annotated screenshot of TREWS evaluation interface: TREWS is a dynamic workflow that updates given context. This screenshot of the interface shows the evaluation page that clinicians completed to assess for sepsis in response to an alert. The evaluation page version shown here is from the research version of the tool that was in use at the time of this study.

Supplementary Table 1. Selected Codes Generated from the Interviews Using Grounded Theory Approach

Code	Description of Code	Example Quotation
**Theme 1: Limited Differentiation of the Operations Behind ML-based and Conventional Clinical Support Systems**		
Mental Models of an ML-based system	Described the model as using rule-based decision logic (e.g., checking if model inputs exceed a pre-specified threshold)	"[The deployed system is] a tool that checks certain clinical parameters that is supposed to detect sepsis or at least early sepsis or to alert you."
Comparison to rule-based CDS	Compared two or more clinical support systems	"That one [rule-based alert] was really irritating because you couldn't do anything. It kept like telling you to reorder lactate so every time you'd open the patient's chart--you'd end up having like fifteen lactic acids from just opening the chart and it was really obnoxious"
**Theme 2: Roles that ML-based Systems Play in Clinical Care**		
**Theme 2.A: Supporting Clinicians in Diagnosis**		
Alert evaluation	Described how they evaluated an alert and determined if they agreed with the alert	"I'm learning I've adapted to it more and more it really is an ongoing assessment tool that doesn't just factor in one piece of information, it's factoring in a gazillion pieces of information."
Role of clinicians	Described providers as having the ultimate authority in diagnosing and treating the patient and does not view the tool as a threat to that	"I see it as all guides, but the final decision is yours."
Clinical intuition	Described clinicians as having intuition that AI could not replicate	"Nurses have instinct and there are visual cues that an [artificial intelligence] could never capture..."<br>"TREWS can't help you with what it can't see."
**Theme 2.B: Assistive Roles Beyond Diagnosis**		
Integrating information	Comments about the tool helping them process information in the EHR	"There's just so much when we get into these charts. It can be overwhelming and you can lose sight of what's important."
Care Coordination	Commented on the extent to which the tool helped them coordinate care	"You can see like what we're required to do... cross our t's and dot our i's."

Patient Prioritization	Commented on the extent to which the tool helped them triage patients	"I think we try to get them in front of a provider a little bit quicker or get some of the stuff started out in triage."
Education	Commented on the extent to which the tool can aid with clinician education about sepsis	"When you have this change constantly with staffing, it...is another identifier for the docs and the nurses."
Monitoring	Described the tool as acting as a backup to help monitor the patients	"I'd call it a second set of eyes and ears for the clinician…Clinical judgment supersedes any tool, but it's there to help you pick up potential septic patients, and that's one component, but the other component is it can help keep you on track with the core measures"
**Theme 3: Mechanisms of Trust**		
Trust Factors	Described trusting the system based on direct evaluation of unprocessed measurements or factors used by the system (e.g., laboratory values and vital signs)	"[T]he extent that I can see all of the factors that are playing a role in the decision, that's helpful to me to trust it. I don't think my understanding has to go beyond that."
Trust Experience	Described trusting the system based on personal experience using it	"I had to test it out and like determine its behaviors."
Trust Evidence	Described trusting the system based on reviewing reported performance characteristics and outcome	"You validate it, you look at the data behind it, you know the research and the validity to the tool."
Trust Peer	Described trusting the system based on peer endorsement	"For the TREWS I know when the score was developed it was researched and [PHYSICIAN NAME] was involved in that like the TREWS was created so there's good data to support that the score is right, the implementation is the next."
Trust Expert	Described trusting the system based on expert recommendation or trust in the developers	"I need to understand the motivation behind that tool because when I apply that tool, I'm applying the judgment of the creators of that tool."
System Feedback	Commented on the extent to which giving feedback to the system impacted their perception of the system	"I don't have a way to tell whoever started this project that, 'Hey, it's capturing too many patients with X'... so nothing's getting fixed. So, in order to trust it, I think I would

		need to be able to give and receive feedback, which we are getting from the TREWS."
Trust Model	Described trusting the system based on a general understanding of the model	"For clinicians, I think just understanding this is a machine learning tool and it does data mining, I think will be more than enough…. I don't think it will make that much of a difference for physicians to go into details."
**Theme 4: Limitations of ML-based Clinical Support Systems**		
Model overreliance	Described concerns about providers becoming over-reliant on automated systems	"I think there are a lot of people frankly that will quickly default to having a tool tell them what to do and stop assessing, and I hope that's not true, but I've seen it happen"
Challenge	Described challenges related to using predictive alerts	"It gets tough to treat something ahead of time, because if someone's doing okay right there right now, if you want to give them a treatment that could potentially cause them harm, that might … give us cause to pause to sort of be pre-emptive about that."
