# Supplementary material for: Human–machine teaming is key to AI adoption: clinicians’ experiences with a deployed machine learning system
Source: NPJ Digit Med. 2022 Jul 21;5:97. doi: 10.1038/s41746-022-00597-7 (PMC9304371; doi:10.1038/s41746-022-00597-7)
Supplement: Supplementary file 2 — Reporting Summary [file 41746_2022_597_MOESM2_ESM.pdf]

nature portfolio

Corresponding author(s): Suchi Saria
Bilge Mutlu

Last updated by author(s): Feb 23, 2022

Reporting Summary

Nature Portfolio wishes to improve the reproducibility of the work that we publish. This form provides structure for consistency and transparency in reporting. For further information on Nature Portfolio policies, see our Editorial Policies and the Editorial Policy Checklist.

Statistics

For all statistical analyses, confirm that the following items are present in the figure legend, table legend, main text, or Methods section.

n/a	Confirmed	
☐	☒	The exact sample size (n) for each experimental group/condition, given as a discrete number and unit of measurement
☒	☐	A statement on whether measurements were taken from distinct samples or whether the same sample was measured repeatedly
☒	☐	The statistical test(s) used AND whether they are one- or two-sided<br>*Only common tests should be described solely by name; describe more complex techniques in the Methods section.*
☒	☐	A description of all covariates tested
☒	☐	A description of any assumptions or corrections, such as tests of normality and adjustment for multiple comparisons
☒	☐	A full description of the statistical parameters including central tendency (e.g. means) or other basic estimates (e.g. regression coefficient) AND variation (e.g. standard deviation) or associated estimates of uncertainty (e.g. confidence intervals)
☒	☐	For null hypothesis testing, the test statistic (e.g. F, t, r) with confidence intervals, effect sizes, degrees of freedom and P value noted<br>*Give P values as exact values whenever suitable.*
☒	☐	For Bayesian analysis, information on the choice of priors and Markov chain Monte Carlo settings
☒	☐	For hierarchical and complex designs, identification of the appropriate level for tests and full reporting of outcomes
☒	☐	Estimates of effect sizes (e.g. Cohen's d, Pearson's r), indicating how they were calculated

Our web collection on statistics for biologists contains articles on many of the points above.

Software and code

Policy information about availability of computer code

Data collection	No software was used for data collection
Data analysis	No software was used for data analysis

For manuscripts utilizing custom algorithms or software that are central to the research but not yet described in published literature, software must be made available to editors and reviewers. We strongly encourage code deposition in a community repository (e.g. GitHub). See the Nature Portfolio guidelines for submitting code & software for further information.

Data

Policy information about availability of data

All manuscripts must include a data availability statement. This statement should provide the following information, where applicable:

- Accession codes, unique identifiers, or web links for publicly available datasets
- A description of any restrictions on data availability
- For clinical datasets or third party data, please ensure that the statement adheres to our policy

The full transcripts of the interviews are not publicly available in order to minimize the risk of participant reidentification. Summaries of the interview contents and related metadata that support these findings are available from the corresponding author upon reasonable request.

Field-specific reporting

Please select the one below that is the best fit for your research. If you are not sure, read the appropriate sections before making your selection.

☐ Life sciences ☒ Behavioural & social sciences ☐ Ecological, evolutionary & environmental sciences

For a reference copy of the document with all sections, see nature.com/documents/nr-reporting-summary-flat.pdf

Behavioural & social sciences study design

All studies must disclose on these points even when the disclosure is negative.

Study description	This is a qualitative study of clinician's perceptions of a data driven clinical support system. The research team conducted semi-structured interviews with clinicians in a hospital using a data driven sepsis alert system. The collected interviews were then transcribed and coded in an iterative process using a Grounded Theory approach to identify themes in the interviews.
Research sample	Study participants included 13 physicians (4 emergency department, 4 critical care unit physicians, 5 general ward) and 7 nurses (3 emergency department, 4 critical care) working at a community hospital in Maryland that used the data driven clinical alert system.
Sampling strategy	Physicians and nurses at the study site who had used the clinical support system (Targeted Real-time Early Warning System for sepsis) for at least six months were asked to participate, thereby representing a number of units and clinical roles. Among clinicians who responded to the investigator's interview requests and were available at the interview times, a representative sample of nurses and physicians across different unit types was selected. Interviews were conducted until saturation was reached, such that conducting new interviews failed to generate novel themes and insights.
Data collection	Interviews were conducted at the hospital, in each participant's work environment (e.g., nurse's stations, private office, etc.). The interviews were conducted by one of two graduate research assistants who were familiar with the system and clinical environment and had been trained in semi-structured interview methods. The interview guide was developed collectively by all authors and questions concerned clinicians' role in diagnosing and treating sepsis, their experience with CDSS in general, their experience with TREWS and other ML-based CDSS, and their thoughts regarding the current and future role of ML in medicine.
Timing	Interviews were conducted between October 2018 and April 2019.
Data exclusions	No data were excluded from the analysis
Non-participation	No participants dropped out or declined participation
Randomization	Participants were not allocated to experimental groups

Reporting for specific materials, systems and methods

We require information from authors about some types of materials, experimental systems and methods used in many studies. Here, indicate whether each material, system or method listed is relevant to your study. If you are not sure if a list item applies to your research, read the appropriate section before selecting a response.

Materials & experimental systems

n/a	Involved in the study	
☒	☐	Antibodies
☒	☐	Eukaryotic cell lines
☒	☐	Palaeontology and archaeology
☒	☐	Animals and other organisms
☐	☒	Human research participants
☒	☐	Clinical data
☒	☐	Dual use research of concern

Methods

n/a	Involved in the study	
☒	☐	ChIP-seq
☒	☐	Flow cytometry
☒	☐	MRI-based neuroimaging

Human research participants

Policy information about studies involving human research participants

Population characteristics	See above.
Recruitment	Physicians and nurses at the study site who had used the data driven clinical support system for at least six months were asked to participate, thereby representing a number of units and clinical roles. Among clinicians who responded to the investigator's interview requests and were available at the interview times, a representative sample of nurses and physicians across different unit types was selected.

Ethics oversight	Johns Hopkins Medicine Internal Review Board reviewed and approved the study protocol (Study protocol number IRB00252594).

Note that full information on the approval of the study protocol must also be provided in the manuscript.
